# Genetic association study of interferon lambda 3*,* CD27*,* and human leukocyte antigen-DPB1 with dengue severity in Thailand

**DOI:** 10.1186/s12879-020-05636-w

**Published:** 2020-12-11

**Authors:** Unchana Arayasongsak, Izumi Naka, Jun Ohashi, Jintana Patarapotikul, Pornlada Nuchnoi, Thareerat Kalambaheti, Areerat Sa-Ngasang, Sumalee Chanama, Suwanna Chaorattanakawee

**Affiliations:** 1 Department of Microbiology and Immunology, Faculty of Tropical Medicine, Mahidol University, Ratchawithi Road, Bangkok, 10400 Thailand; 2 Laboratory of Human Genome Diversity, Department of Biological Sciences, Graduate School of Science, The University of Tokyo, Hongo, Bunkyo-ku, Tokyo, 113-0033 Japan; 3 Department of Clinical Microscopy, Faculty of Medical Technology, Mahidol University, Bangkok, 10700 Thailand; 4 National Institute of Health, Department of Medical Sciences, Ministry of Public Health, Nonthaburi, Thailand; 5 Department of Parasitology and Entomology, Faculty of Public Health, Mahidol University, Ratchawithi Road, Bangkok, 10400 Thailand

**Keywords:** IFNL3, CD27, HLA-DPB1, Dengue, Genetic association, Disease severity

## Abstract

**Background:**

Dengue patients develop different disease severity ranging from mild (dengue fever [DF]) to severe forms (dengue hemorrhagic fever [DHF] and the fatal dengue shock syndrome [DSS]). Host genetics are considered to be one factor responsible for the severity of dengue outcomes. To identify genes associated with dengue severity that have not been studied yet, we performed genetic association analyses of interferon lambda 3 (IFNL3)*,* CD27*,* and human leukocyte antigen-DPB1 (HLA-DPB1) genes in Thai dengue patients.

**Methods:**

A case–control association study was performed in 877 children (age ≤ 15 years) with dengue infection (DF, *n* = 386; DHF, *n* = 416; DSS, *n* = 75). A candidate single nucleotide polymorphism of each of *IFNL3, CD27,* and *HLA-DPB1* was selected to be analyzed. Genotyping was performed by TaqMan real-time PCR assay, and the association with dengue severity was examined.

**Results:**

The rs9277534 variant of *HLA-DPB1* was weakly associated with DHF. The genotype GG and G allele conferred protection against DHF (*p* = 0.04, odds ratio 0.74 for GG genotype, *p* = 0.03, odds ratio 0.79 for G allele). The association became borderline significant after adjusting for confounders (*p* = 0.05, odds ratio 0.82). No association was detected for *IFNL3* or *CD27*.

**Conclusions:**

The present study demonstrated the weak association of the rs9277534 variant of *HLA-DPB1* with protection against DHF. This variant is in the 3′ untranslated region and affects HLA-DPB1 surface protein expression. Our finding suggests that HLA-DPB1 may be involved in DHF pathogenesis.

## Background

Dengue is a major life-threatening disease and might be the most common mosquito-borne viral disease in tropical and subtropical regions. Dengue infection causes a wide range of clinical presentations from mild (dengue fever [DF]) to severe (dengue hemorrhagic fever [DHF] and the fatal dengue shock syndrome [DSS]). Pathogenic factors that determine differences in the clinical manifestations of dengue infection are not well understood but evidence suggests that severe disease is associated with secondary infection, virulent virus serotype, and host genetic factors [1–3]. Many studies reported that antibody-dependent enhancement of secondary dengue virus infection is a key factor of DHF/DSS [4]. However, it does not fully explain differences in severity in other cases.

Regarding human genetics, individuals with European ancestry apparently develop severe dengue more frequently than those with African ancestry [5, 6]. This observation suggests that human gene polymorphisms may contribute to susceptibility to developing severe symptoms. To date, a number of candidate gene variants associated with dengue severity have been identified, many of which are involved in the immune system, indicating the immune response of patients may impact disease severity. Nonetheless, the pathogenicity of severe dengue is complex, involving several immune cell types, cytokines, and other immunoregulatory molecules, as well as several genetic variants that affect the disease outcome. To obtain complete knowledge of genes/markers that predict dengue outcomes, we investigated genes that have not been studied to date, interferon lambda 3 (*IFNL3*)*, CD27,* and human leukocyte antigen-DPB1 *(HLA-DPB1)*. A candidate single nucleotide polymorphism (SNP) of each gene was selected to be analyzed, and its association with dengue severity in Thailand was examined.

Interferon lambda 3 (IFNL3), also known as interleukin 28B, is a member of the type III Interferons (IFNs). The *IFNL3* gene is encoded on chromosome 19 [7]. The function of type III IFNs is similar to that of type I IFNs (IFN-α and IFN-β) induced by viral infections and includes antiviral activity [8]. Several SNPs in *IFNL3* and its upstream and downstream regions were associated with the spontaneous clearance of hepatitis C virus and the response to therapy [8–14]. Among those markers, rs8099917 was the most common relevant SNP and was associated with the mRNA expression of *IFNL3* in the liver and peripheral blood mononuclear cells [9, 12]. The rs8099917 was also analyzed in dengue patients in Mexico, but showed no association with development of DHF [15]. Therefore, rs8099917 located 8.9 kb upstream of IFN-λ3 was analyzed here for its association with dengue severity in Thai population.

CD27 is a member of the tumor necrosis factor superfamily. The expression of CD27 is restricted to lymphocytes. CD27 activates nuclear factor kappa B signaling in T cells [16]. Soluble forms of CD27 (sCD27) are present in body fluids such as plasma, and plasma levels of sCD27 have been used to monitor and track viral infections [17, 18]. Of note, plasma levels of sCD27 were higher in dengue-infected children than in healthy children, and in severe dengue compared with mild dengue [19]. Although *CD27* polymorphisms associated with infectious diseases have not been identified, rs2267966 was reported to be associated with a decreased risk of breast cancer in a northern Chinese population [20]. Therefore, rs2267966 was selected for association analysis with dengue severity.

Human leucocyte antigen (HLA) is encoded by the major histocompatibility complex on chromosome 6, which is the most polymorphic in the human genome [21]. HLA class I (HLA-A, −B, −C) and HLA class II (HLA-DR, −DQ, and -DP) molecules are involved in presenting antigen to T cells, which lead to anti-viral responses [22]. Although several studies demonstrated the genetic association of HLA with dengue disease [23–27], the role of *HLA-DP* in dengue severity has not been studied yet. Previous studies reported the association of *HLA-DPB1* with the spontaneous clearance of hepatitis B virus (HBV) in Japanese and American populations [28, 29], and susceptibility to systemic sclerosis (SSc) in Korean and North American populations [30]. Therefore, we selected rs9277534, a SNP in the 3′ untranslated region (UTR) associated with HLA-DPB1 expression, [29], to determine its association with dengue severity.

This study analyzed the rs8099917 of *IFNL3*, rs2267966 of *CD27,* and rs9277534 of *HLA-DPB1* for their association with dengue severity in a Thai population.

## Methods

### Subjects and definition of dengue fever

Candidate gene analysis based on a case-control association study was performed to investigate the genetic association of *IFNL3, CD27,* and *HLA-DPB1* genes with dengue severity. We thus included 877 patients with DF (*n* = 386), DHF (*n* = 416), or DSS (*n* = 75). Patients with DF who develop only acute febrile illness are considered as the control groups, whereas those who develop severe and fetal complications (DHF and DSS) are considered as the case groups. The SNPs rs8099917 of *IFNL3,* rs2267966 of *CD27*, and rs9277534 of *HLA-DPB1* were selected to be analyzed. DNA samples were collected from dengue patients hospitalized in Ratchaburi and Lampang hospital, Thailand during 2000 to 2004. To reduce potential bias due to a difference in immunity among different ages groups, all patients participated in this study were children aged ≤15 years old. Dengue virus infection was confirmed in all patients by capture enzyme-linked immunosorbent assay for IgM and IgG, reverse transcriptase polymerase chain reaction, and/or dengue virus isolation in a C6/36 cell line at the Arbovirus Laboratory, National Institute of Health, Department of Medical Sciences, Ministry of Public Health, Thailand. The patients were categorized into three groups: DF, DHF, and DSS according to World Health Organization 1997 criteria. The characteristics of patients (age, sex, sample collecting regions, dengue virus serotype, and dengue immune status of patients) in each group were described previously [31], and the comparison among the groups was summarized in Table 1. The result showed significant differences in sample collecting regions, dengue virus serotype, and immune status of patients among the groups. DF was characterized by a febrile disease with various nonspecific symptoms such as severe headache, myalgia, arthralgia, rashes, and leucopenia. DHF was defined by the additional criteria including hemorrhagic manifestations such as petechiae, plasma leakage as shown by ≥20% increase in hematocrit from baseline, and thrombocytopenia (platelet count ≤100,000/μL). DSS, the most severe form of dengue infection, was characterized by all the criteria of DHF with an addition sign of shock manifested by a rapid and weak pulse with narrowing pulse pressure (≤ 20 mmHg) or hypotension with cold, clammy skin, and restlessness [32]. The study was reviewed and approved by the Institutional Review Board of the Faculty of Tropical Medicine, Mahidol University and Department of Biological Sciences, the Graduate School of Science, the University of Tokyo.

**Table 1:** Summary of demographical data, dengue virus serotype, and immune status of patients with dengue fever (DHF), dengue hemorrhagic fever (DHF), and dengue shock syndrome (DSS) in the association study

Characteristics	Number of subject (%)			*P*-value^c^		
	DF (*n* = 386)	DHF (*n* = 416)	DSS (*n* = 75)	DF vs DHF	DF vs DSS	DHF vs DSS
Age: Median (IQR)	9 (7–12)	10 (7–12)	9 (7–12)	0.025	0.888	0.257
Sex:						
Male	200 (51.81)	223 (53.61)	40 (53.33)	0.611	0.809	0.965
Female	186 (48.19)	193 (46.39)	35 (46.67)			
Region^a^:						
Lampang	166 (43.01)	108 (26.02)	24 (32.00)	< 0.001	0.076	0.28
Ratchaburi	220 (56.99)	307 (73.98)	51 (68.00)			
Dengue virus serotype^b^:						
DENV-1	142 (36.79)	130 (31.32)	15 (20.00)	0.007	< 0.001	0.005
DENV-2	102 (26.42)	152 (36.63)	39 (52.00)			
DENV-3	89 (23.06)	71 (17.11)	5 (6.67)			
DENV-4	53 (13.73)	62 (14.94)	16 (21.33)			
Immune status:						
1^o^ infection	72 (18.7)	47 (11.3)	0 (0)	0.003	< 0.001	0.002
2^o^ infection	314 (81.3)	369 (88.7)	75 (100)			

^a^ A patient with unidentified region was not shown in the table

^b^ A patient infected with mixed dengue virus serotype was not shown in the table

^c^ *P*-values of χ2 (for qualitative variables) and Mann-Whitney U test (for ages) were shown

### SNP genotyping

Genomic DNA from 877 patients was extracted from buffy coat using a QIAamp DNA blood mini kit (Qiagen, Hilden, Germany). The protocol for DNA extraction as described by the manufacturer’s instructions was processed on a QIAvac24 (Qiagen).

TaqMan SNP analysis kits, assay ID C__11710096_10, C__15873426_10, and C__29841700_20 (Applied Biosystems, Foster City, CA, USA), were used for the genotyping of rs8099917, rs2267966, and rs9277534, respectively, following the manufacturer’s procedure (Applied Biosystems). The context sequences of rs8099917, rs2267966, and rs9277534 are shown in Table 2. A standard thermal cycler was used to conduct polymerase chain reaction (PCR) with condition as follows: pre-denaturation at 95 °C for 60 s, followed by 40 cycles of amplification at 95 °C for 15 s and 60 °C for 30 s. Endpoint PCR products were analyzed in a 7300 Real-time PCR system (Applied Biosystems). Fluorescence signal were plotted and the allele was determined using ABI sequence detection 7300 SDS software (Version 1.3.1).

**Table 2:** Context sequences of SNPs analyzed in this study

Gene	SNPs	Context Sequences^a^
*IFNL3*	rs8099917	TTTTGTTTTCCTTTCTGTGAGCAAT [G/T] TCACCCAAATTGGAACCATGCTGTA
*CD27*	rs2267966	TTCTTCCCTTGCAGGTCCTTATCCA [A/T] CATGGCTCACAATTCCTGGGAAGCC
*HLA-DPB1*	rs9277534	CCCTCATCCATTTATGTCTCAGACC [A/G] CTATTCTTAACTATTCAATGGTGAG

^a^The G and T variants of rs8099917, A and T variants of rs2267966, and A and G variants of rs9277534 were detected by VIC and FAM fluorescence signals, respectively

### Statistical analysis

The deviation of genotype frequency from Hardy-Weinberg equilibrium was evaluated in each group of dengue patients (DF, DHF and DSS) via an HWE web tool (http://www.oege.org/software/hwe-mr-calc.shtml) [33]. Stata version 14 (Stata Corp., TX, USA) was used to examine the association of rs8099917 in *IFNL3*, rs2267966 in *CD27,* and rs9277534 in *HLA-DPB1* with dengue severity. Chi-squared test was performed to compare the genotype and allele frequencies in the DF, DHF, and DSS groups, and odds ratios (OR) was calculated. The Fisher’s exact test was used when one or more cell in 2 × 2 contingency table contained the expected values of less than five. MedCalc for Windows version 12.7.7.0 (MedCalc Software, Ostend, Belgium) was used to determine OR and 95% confidence interval (CI) when a cell value in a contingency table equals to zero. Confounding factors including age, sex, region, dengue virus serotype, and primary/secondary infection were adjusted by a multiple logistic regression analysis using R program version 3.4.1. Statistical significance was defined when *p* < 0.05.

## Results

Of 877 samples, genotyping was success in 871 (99.3%), 866 (98.7%), and 873 (99.5%) for rs8099917 (*IFNL3*), rs2267966 (*CD27*), and rs9277534 (*HLA-DPB1*) respectively. The genotype and allele frequencies of rs8099917 (*IFNL3*) and rs2267966 (*CD27*) are shown in Tables 3 and 4, respectively. They were not significantly different between dengue fever (DF), dengue hemorrhagic fever (DHS), and dengue shock syndrome (DSS) groups, suggesting the rs8099917 of *IFNL3* and rs2267966 of *CD27* were not associated with the pathogenesis of DHF and DSS in Thai patients. For rs9277534 of *HLA-DPB1* (Table 5), there was no difference in genotype frequencies between DF and DHF. However, when allele frequencies were analyzed, the frequency of the G allele was significantly lower in DHF than in DF (61.0% vs 66.3%), with a *p*-value of χ^2^ = 0.03, suggesting the G allele was associated with a reduced risk of DHF (OR = 0.79; 95% CI = 0.64–0.98). Accordingly, considering a recessive model, the group of patients with the G/G genotype were apparently protected against DHF, relative to those with A/A and A/G (*p*-value of χ^2^ = 0.04, OR = 0.74; 95% CI = 0.55–0.99). We performed a multiple logistic regression analysis to confirm the association by adjusting for the effects of confounding factors including age, sex, region, dengue immune status and virus serotype. After adjustment, the association became borderline significant (*p*-value = 0.05, OR = 0.82; 95% CI = 0.66–1.00). There was no difference in allele and genotype frequencies between DS and DSS and between DHS and DSS.

**Table 3:** Association analysis of the *IFNL3* polymorphism (rs8099917) with dengue severity in Thailand

Genotype /allele	Number of subjects			DF vs DHF		DF vs DSS		DHF vs DSS	
	DF (*n* = 384)	DHF (*n* = 413)	DSS (*n* = 74)	*P*-value^a^	OR (95%CI)	*P*-value^a^	OR (95%CI)	*P*-value^a^	OR (95%CI)
T/T	342 (89.1%)	365 (88.4%)	67 (90.5%)	0.95	1	1.00	1	1.00	1
G/T	38 (9.9%)	43 (10.4%)	7 (9.5%)		1.06 (0.65–1.73)		0.94 (0.34–2.25)		0.88 (0.32–2.10)
G/G	4 (1.0%)	5 (1.2%)	0 (0%)		1.17 (0.25–5.95)		0.56 (0.03–10.59)		0.49 (0.03–9.01)
T	722 (94.0%)	773 (93.6%)	141(95.3%)	0.72	1	0.55	1	0.43	1
G	46 (6%)	53 (6.4%)	7 (4.7%)		1.07 (0.70–1.65)		0.78 (0.29–1.78)		0.72 (0.27–1.64)
**Dominant Model**									
T/T	342 (89.1%)	365 (88.4%)	67 (90.5%)	0.76	1	0.71	1	0.59	1
G/T, G/G	42 (10.9%)	48 (11.6%)	7 (9.5%)		1.07 (0.67–1.70)		0.85 (0.31–2.02)		0.79 (0.29–1.86)
**Recessive Model**									
T/T, G/T	380 (99.0%)	408 (98.8%)	74 (100%)	1.00	1	1.00	1	1.00	1
G/G	4 (1.0%)	5 (1.2%)	0 (0%)		1.16 (0.25–5.91)		0.57 (0.03–10.65)		0.49 (0.03–9.11)
**Overdominant Model**									
T/T, G/G	346 (90.1%)	370 (89.6%)	67 (90.5%)	0.81	1	0.91	1	0.80	1
G/T	38 (9.9%)	43 (10.4%)	7 (9.5%)		1.06(0.65–1.73)		0.95 (0.34–2.28)		0.89(0.32–2.13)

^a^*P*-values of χ2 or Fisher’s exact test are shown

**Table 4:** Association analysis of the *CD27* polymorphism (rs2267966) with dengue severity in Thailand

Genotype /allele	Number of subjects			DF vs DHF		DF vs DSS		DHF vs DSS	
	DF (n = 384)	DHF (*n* = 409)	DSS (*n* = 73)	*P*-value^a^	OR (95%CI)	*P*-value^a^	OR (95%CI)	*P*-value^a^	OR (95%CI)
A/A	168 (43.8%)	187 (45.7%)	30 (41.1%)	0.66	1	0.19	1	0.28	1
A/T	165 (42.9%)	176 (43.0%)	38 (52.1%)		0.96 (0.70–1.30)		1.29 (0.74–2.26)		1.35 (0.77–2.35)
T/T	51 (13.3%)	46 (11.3%)	5 (6.8%)		0.81 (0.50–1.30)		0.55 (0.16–1.54)		0.68 (0.19–1.90)
A	501 (65.2%)	550 (67.2%)	98 (67.1%)	0.39	1	0.66	1	0.98	1
T	267 (34.8%)	268 (32.8%)	48 (32.9%)		0.91 (0.74–1.13)		0.92 (0.62–1.36)		1.01 (0.68–1.48)
**Dominant Model**									
A/A	168 (43.8%)	187 (45.7%)	30 (41.1%)	0.58	1	0.67	1	0.46	1
A/T, T/T	216 (56.2%)	222 (54.3%)	43 (58.9%)		0.92 (0.69–1.23)		1.11 (0.65–1.92)		1.21 (0.71–2.08)
**Recessive Model**									
A/A, A/T	333 (86.7%)	363 (88.7%)	68 (93.2%)	0.38	1	0.12	1	0.26	1
T/T	51 (13.3%)	46 (11.3%)	5 (6.8%)		0.83 (0.53–1.29)		0.48 (0.14–1.26)		0.58 (0.17–1.53)
**Overdominant Model**									
A/A, T/T	219 (57.0%)	233 (57.0%)	35 (47.9%)	0.98	1	0.15	1	0.15	1
A/T	165 (43.0%)	176 (43.0%)	38 (52.1%)		1.00 (0.74–1.34)		1.44 (0.84–2.46)		1.43 (0.84–2.44)

^a^*P*-values of χ2 or Fisher’s exact test are shown

**Table 5:** Association analysis of the *HLA-DPB1* polymorphism (rs9277534) with dengue severity in Thailand

Genotype /allele	Number of subjects			DF vs DHF		DF vs DSS		DHF vs DSS	
	DF (n = 386)	DHF (n = 413)	DSS (n = 74)	*P*-value^a^	OR (95%CI)	*P*-value^a^	OR (95%CI)	*P*-value^a^	OR (95%CI)
A/A	47 (12.2%)	64 (15.5%)	6 (8.1%)	0.09	1	0.43	1	0.25	1
A/G	166 (43.0%)	194 (47.0%)	37 (50.0%)		0.86 (0.54–1.35)		1.75 (0.67–5.36)		2.03 (0.79–6.16)
G/G	173 (44.8%)	155 (37.5%)	31 (41.9%)		0.66 (0.42–1.04)		1.40 (0.53–4.36)		2.13 (0.82–6.54)
A	260 (33.7%)	322 (39.0%)	49 (33.1%)	**0.03**	1	0.89	1	0.18	1
G	512 (66.3%)	504 (61.0%)	99 (66.9%)		**0.79 (0.64–0.98)**		1.03 (0.69–1.52)		1.29 (0.88–1.91)
**Dominant Model**									
A/A	47 (12.2%)	64 (15.5%)	6 (8.1%)	0.18	1	0.32	1	0.09	1
A/G, G/G	339 (87.8%)	349 (84.5%)	68 (91.9%)		0.76 (0.49–1.16)		1.57 (0.63–4.67)		2.08 (0.85–6.10)
**Recessive Model**									
A/A, A/G	213 (55.2%)	258 (62.5%)	43 (58.1%)	**0.04**	1	0.64	1	0.48	1
G/G	173 (44.8%)	155 (37.5%)	31 (41.9%)		**0.74 (0.55–0.99)**		0.89 (0.52–1.51)		1.20 (0.69–2.04)
**Overdominant Model**									
A/A, G/G	220 (57.0%)	219 (53.0%)	37 (50.0%)	0.26	1	0.27	1	0.63	1
A/G	166 (43.0%)	194 (47.0%)	37 (50.0%)		1.17 (0.87–1.56)		1.32 (0.78–2.25)		1.13 (0.67–1.91)

^a^*P*-values of χ2 or Fisher’s exact test are shown and significant values are shown in bold

The Hardy-Weinberg Equilibrium test for rs8099917 (*IFNL3*) showed that its genotype frequency in DF and DHF was significantly deviated from Hardy-Weinberg Equilibrium (*p* = 0.02 and *p* = 0.01, respectively), but was in equilibrium in DSS (*p*-value of 0.69). The genotype frequencies of rs2267966 (*CD27*) and rs9277534 (*HLA-DPB1*) in the DF, DHF, and DSS groups were in equilibrium (rs2267966; *p* = 0.30, 0.64, and 0.13, respectively and rs9277534; *p* = 0.40, 0.80, and 0.27, respectively).

## Discussion

We did not find an association of *IFNL3* rs8099917 or CD27 rs2267966 with dengue severity in a Thai population, whereas *HLA-DPB1* rs9277534 showed a weak association. The G allele of rs9277534 was associated with a reduced risk of DHF compared with the A allele. However, this association became borderline significant when multiple logistic regression analysis was performed to adjust for confounding factors. Our study suggests that *IFNL3* and CD27 may not be involved in the pathogenesis of DHF and DSS. The G allele of *IFNL3* rs8099917 (genotype TG and GG) was associated with a lower mRNA expression of *IFNL3* [9, 34], but our results did not reveal an effect of this SNP on dengue severity. However, to date very few SNPs of *IFNL3* have been studied, and therefore further studies are required to determine the role of *IFNL3* in dengue infection. The role of CD27 in dengue severity is also unclear. Although *CD27* rs2267966 was associated with a risk of breast cancer [20], its function is unknown and we did not find an association with dengue severity. Future studies on other SNPs of CD27 are required because the plasma level of soluble CD27 was higher in severe dengue than in mild dengue [19]. Nonetheless, a limitation of this study could be a low sample size of DSS (*N* = 75) relating to its incidence. This could reduce the power to detect risk markers, and their effect on disease progression to DSS.

Evidence suggesting human genetics are responsible for dengue severity was obtained by HLA serotyping. Here, we demonstrated at the gene level that *HLA-DPB1* rs9277534 was weakly associated with DHF. *HLA-DPB1* is a gene that encodes HLA class II antigens. The HLA-DP molecule is expressed on the surface of antigen presenting cells that present extracellular antigens to CD4^+^ T cells to stimulate immune responses. Genome-wide studies showed an association of *HLA-DPB1* with chronic hepatitis B virus (HBV) infection in Asians [28, 35–38]. Moreover, two SNPs in the 3′UTR region of *HLA-DPB1*, rs9277534 and rs9277535, were associated with hepatitis B viral clearance in those of European-American or African-American ancestry, [29] as well as Asians [28]. The GG genotype of rs9277534 was associated with increased HLA-DP expression, and conferred susceptibility to HBV persistent infection. It was hypothesized that a high expression of HLA-DP surface protein favors a Th2 response, a poor Th1 response, and cytotoxic T cell lymphocyte activity, resulting in HBV persistence [29]. An imbalance in the Th1/Th2 response may also explain the association of HLA-DPB1 with Th1-mediated rheumatoid arthritis [29, 39, 40]. However, the role of HLA-DP in dengue pathogenesis has not been described. In the present study, weak association of the rs9277534 GG genotype with protection against DHF would support a contribution of HLA-DP and immune imbalance in DHF pathogenesis. Nonetheless, studies to identify other genetic loci or key molecules underlying DHF are required. Previous studies reported distinct genetic markers for risk of DHS and DSS, implying separate pathogenic processes in these two dengue outcomes. Therefore, identifying susceptible/protective SNPs for DHF and DSS will help predict dengue outcomes accurately.

## Conclusions

This is the first report of the genetic association of *IFNL3, CD27,* and *HLA-DPB1* with dengue outcomes. Although one candidate SNP of each gene was analyzed, and no significant association was observed for *IFNL3* and *CD27*, further analysis of other gene variants is warranted to determine their role in dengue severity. Interestingly, we found a 3′ UTR variant of *HLA-DPB1* (rs9277534), which has been proved to increase HLA-DPB1 surface expression, was weakly associated with protection from DHF in Thailand. This finding suggests HLA-DP might be involved in DHF pathogenesis.

## Data Availability

The data sharing of research project under Institutional Review Board approval (the Faculty of Tropical Medicine, Mahidol University) is not subjected to be allowed currently. However, it could be available upon request.

## Abbreviations

CI: Confidence interval
DF: Dengue fever
DHF: Dengue hemorrhagic fever
DSS: Dengue shock syndrome
HBV: Hepatitis B virus
HLA: Human leucocyte antigen
HLA-DPB1: Human leukocyte antigen-DPB1
IFN (s): Interferon (s)
IFNL3: Interferon lambda 3
OR: Odds ratio
PCR: Polymerase chain reaction
sCD27: Soluble forms of CD27
SNP (s): Single nucleotide polymorphism (s)
UTR: Untranslated region
